# Delay

**Published:** 1867-10

**Authors:** 


### Editorial.

### *Delay.*

The present Number of the JOURNAL has been delayed a few days, in order to present to our readers a full account of the proceedings introductory to the TWENTY-FIFTH Annual Session of Rush Medical College. This event, under all the existing circumstances, we consider one of the most important in the medical history of the North-west. It inaugurates a new era, and should be hailed, by all who have the real welfare of the profession at heart, with cordial congratulations.

This rapidly expanding, great, and enterprising metropolis has now an edifice devoted to medical science, commensurate with the demands of the time. It is no mere boast to say that the continent cannot show its equal, whether we refer to its magnitude, architectural beauty, or general adaptation, in all its parts and appointments, for its designed purpose.

It is gratifying to be able to add that the opening exercises, the meeting of the Alumni, the concise history of the College given by President Blaney, the appropriate remarks by His Honor, Mayor Rice, the splendid oration by Prof. Gunn, who brings additional prestige to the chair before rendered illustrious by the lamented Brainard, the pleasant reunion of the Faculty, Alumni, students, and profession, which followed—all agreeable to the utmost of desire, have proven not vain formalities, for the College now seats in its ample halls a larger class than ever before convened in any strictly legitimate medical college west of the Atlantic cities. This effort, as all real efforts will, has achieved its legitimate reward.

From the editorial knowledge of the Faculty, we predict a success in medical teaching, the present and future sessions, heretofore unparalleled. *Nous verrons.*

